# Vaccination Update and Specific Concerns for RA

**DOI:** 10.1007/s11926-025-01197-6

**Published:** 2025-09-17

**Authors:** Mariana Urquiaga, Kevin L. Winthrop, Jeffrey R. Curtis

**Affiliations:** 1 Division of Clinical Immunology and Rheumatology, University of Alabama at Birmingham, Birmingham, AL USA; 2 School of Public Health, Oregon Health & Science University-Portland State University, Portland, OR USA; 3 Division of Infectious Diseases, Oregon Health & Science University, Portland, OR USA

**Keywords:** Immunogenicity, Reactogenicity, Shingles, Measles, MMR, RSV

## Abstract

**Purpose of Review:**

We present information on the burden of vaccine-preventable diseases in people with rheumatoid arthritis (RA), the latest evidence on vaccine immunogenicity in disease-modifying antirheumatic drug (DMARD) users, and expert and guideline-based immunization recommendations. We focus on infections with the highest morbidity and mortality, and those relevant due to new developments or current outbreaks.

**Recent Findings:**

Following the license expansion for two respiratory syncytial virus (RSV) vaccines, GSK’s Arexvy and Pfizer’s Abrysvo, the Advisory Committee for Immunization Practices (ACIP) expanded the recommendation for vaccination in adults at increased risk of severe RSV disease. In the spring of 2025, the Center for Disease Control lowered the cutoff for immunization in high-risk groups from ≥ 60 to ≥ 50 years. There are new 2024–2025 SARS-CoV-2 vaccines and updated ACIP recommendations for SARS-CoV-2 immunization that address new viral strains and the known waning immunity from vaccines. All individuals who are moderately to severely immunocompromised (including those with RA) should receive at least one additional vaccine dose compared to the general population. The ACIP has updated its recommendations for pneumococcal immunization, aiming to lower pneumococcal disease incidence in adults. Following the approval of the 21-valent pneumococcal conjugate vaccine, designed to target the serotypes commonly affecting adults, the cutoff for vaccination in the general population changed from ≥ 65 to ≥ 50 years. Recommendations for vaccination in RA patients (everyone age ≥ 18 years) remain unchanged.

**Summary:**

Vaccine recommendations for RA patients constantly evolve as new DMARDs and vaccines are developed, and our understanding of their interaction with DMARDs vis a vis immunogenicity improves. It is essential to stay current with the latest recommendations from the ACIP and rheumatologic society guidelines.

## Introduction

People with rheumatoid arthritis (RA) are at increased risk of acquiring infections and having greater infection-related morbidity and mortality, particularly those who are older, have comorbidities, and are using long-term glucocorticoids and certain immunosuppressive drugs [1]. Vaccines can reduce these risks. However, their use in people with RA poses special considerations regarding the interaction between the underlying disease, the disease-modifying antirheumatic drugs (DMARDs) that patients are receiving, vaccine efficacy, and potential vaccine-related effects. Not surprisingly, there is confusion and concern about the use of vaccines, and overall, vaccines are underutilized in RA patients.

Given the burden of vaccine-preventable diseases and their complications in people with RA, it is important to have clear vaccine indications and administration guidelines. When developing recommendations and making decisions for their patients, clinicians must weigh the disease burden of vaccine-preventable infections and the vaccine’s mechanism of action against the mechanisms and effects of DMARDs that influence vaccine immunogenicity.

This review gathers the latest evidence on the epidemiology of vaccine-preventable infections, describes the effectiveness, safety, and considerations of various vaccines in patients with RA or those using therapies commonly used by RA patients, and provides updated recommendations and guidance regarding the optimal use of vaccines in individuals with RA. It aims to provide readers with comprehensive and up-to-date evidence and share personal insights that may facilitate personalized treatment and shared decision-making.

### Vaccine-Preventable Diseases and their Complications in People with RA

Infections pose a significant risk to individuals with RA, a group with a higher prevalence of comorbidities than the general population and one that suffers from an altered immune system due to the disease and the immunosuppressive therapies commonly used for its management [2]. Patients with RA are more susceptible to certain infections, like herpes zoster reactivation and pneumonia, than the general population [3, 4]. In addition to a higher likelihood of acquiring infections, the presence of comorbidities and the use of immunosuppressive drugs can compromise patients’ response to infections, making complications like hospitalizations, the need for critical care, and death more likely [5]. This review will focus on the pathogens with the largest burden in people with RA and provide relevant and recent updates related to new vaccines (i.e., respiratory syncytial virus), new evidence for existing vaccines (e.g., herpes zoster), or in light of recent outbreaks (i.e., measles).

### Respiratory Infections

Respiratory infections are the most common serious infections - those requiring hospitalization or intravenous antibiotics - in people with RA [6-9]. In a study evaluating the incidence of serious infections in tumor necrosis factor (TNF)-inhibitor (TNFi) initiators from the Consortium of Rheumatology Researchers of North America (Corrona) RA registry, the overall rate of serious infections was 6–7 per 100 person-years (PY), and pneumonia was the most frequently reported infection (together with cellulitis and skin infections and urinary tract infections) [6]. After adjustment (through propensity score matching), there were no differences between TNFi therapies. In two studies using data from the British Society for Rheumatology Biologics Register– RA (BSRBR-RA), respiratory infections were the most common serious infection in the overall RA population (annual IR for an index serious infection of 2.30 [95% CI: 2.22, 2.38]) and in the subset of patients treated with biologic DMARDs (bDMARDs) [42% of all SI] [7, 8]. A study using data from the Norwegian-Disease Modifying Antirheumatic Drug (NOR-DMARD) registry, which includes patients with RA and psoriatic arthritis, showed similar results, and found an overall rate of serious infections of 4.17 (95% CI 3.52, 4.95); 37% of all serious infections were respiratory infections [9].

While some studies reporting respiratory infections in RA patients do not identify the specific pathogen, among those that do, many of the organisms causing respiratory infections in people with RA are targeted by available vaccines. In a study of 148 patients with RA-associated interstitial lung disease (RA-ILD), the most common pathogens causing respiratory infections were SARS-CoV-2 (COVID-19), *Streptococcus pneumoniae*, influenza A virus, and *Pseudomonas aeruginosa* [10]. A larger study conducted in Japan of 1549 patients with pneumonia had similar results, finding that *S. pneumoniae*, *P. aeruginosa*, *Haemophilus influenza*, *Mycoplasma pneumoniae*, and influenza virus were the most common respiratory pathogens in patients with RA [11]. Of these, *S. pneumoniae*, *H. influenza*, COVID-19, and influenza virus are potentially vaccine-preventable. Several studies evaluating hundreds to thousands of participants, conducted in Europe and the United States (US), have found a higher risk of influenza infection, streptococcal pneumonia, and COVID-19 infection in people with RA compared to healthy controls [12–14]. Patients with RA also appear to have a higher risk of polymicrobial respiratory infections. The study conducted in Japan cited above found that people with RA have a three-fold higher risk of polymicrobial pneumonia compared to people without RA [11].

Respiratory syncytial virus (RSV) is known for causing respiratory infections in infants and young children. However, it can also cause severe respiratory illness in older adults and those with cardiopulmonary comorbidities and immunocompromising conditions [15]. In a study of 188 patients with RMD and confirmed RSV infection, older age and a concurrent infection were associated with hospitalization. There was also a trend suggesting increased risk of RSV among users of anti-CD20 therapy and people with interstitial lung disease (ILD). Of the 23 patients with ILD, 17 were hospitalized, 12 died within 90 days, and eight deaths were attributed to RSV [15]. This is one of the few studies evaluating RSV in individuals with RMD or those taking immunosuppressive treatments. In fact, most of the knowledge regarding the risk for severe RSV infection originates from studies of older adults with cardiopulmonary comorbidities rather than in patients with RA or other immunocompromising conditions. Expanding the epidemiology of RSV in RA and other immune mediated inflammatory diseases remains an important evidence gap necessary to address now that vaccination is available.

### Herpes Zoster (shingles)

The incidence of herpes zoster (HZ), caused by the reactivation of the varicella zoster virus (VZV), is significantly higher in patients with RA compared to non-RA controls. In a study using data from the Taiwan National Health Insurance Research Database, the incidence of HZ in RA patients was 21.95 events per 1000 PY, compared to 7.36 events per 1000 PY in the matched controls without RA [16]. A second cohort from the US found very similar results and reported an incidence of HZ in RA patients of 21.5 events per 1000 PY, nearly three times higher than in the non-RA cohort [4]. Patients taking Janus kinase inhibitors (JAKi) and prolonged glucocorticoids appear to have the highest risk of infection among patients with RA [16, 17].

### Human Papillomavirus Infection

People with RA are at an increased risk of persistent human papillomavirus (HPV) infection and subsequent cervical dysplasia and cervical cancer, mainly because immunosuppression impairs the ability to clear HPV infections [18]. In the Million Women Study, an extensive study of women invited for routine breast screening at National Health Service (NHS) screening centers in England and Scotland, RA was associated with a risk of cervical cancer (hazard ratio [HR] 1.39, 95% CI: 1.11, 1.75) that was equivalent to the risk of lymphoid (HR 1.25, 95% CI 1.18, 1.33) and lung cancer (HR 1.21, 95% CI 1.15, 1.26): the two cancers that are most commonly reported to be more likely in people with RA than the general population [19].

### Measles

On March 7, 2025, the Centers for Disease Control and Prevention (CDC) issued a Health Alert Network Health Advisory to notify of a measles outbreak in Texas and New Mexico [20]. As of that date, 208 confirmed cases and two deaths were reported. As of May 1, 2025, 935 measles cases have been confirmed in the US, of which 869 are outbreak-associated [21]. One measles case is estimated to result in 12 to 18 secondary cases in a fully susceptible population, making the measles virus one of the most highly contagious human pathogens known [22].

Measles infection in immunocompromised individuals can lead to severe complications, including pneumonia, encephalitis, and subacute sclerosing panencephalitis, which can be life-threatening [23]. Though there are no studies directly evaluating measles infection in people with RA, in a study of 161 patients with measles who were hospitalized during a 2018 outbreak in Israel, 12 were immunocompromised from a variety of conditions, including the use of immunosuppressive drugs [24]. Of them, two died of measles-related complications. In a similar study done during an outbreak in France that included 36 adults admitted to an intensive care unit for measles complications, seven were immunosuppressed, three were receiving corticosteroids, and five people died– all of them immunocompromised [25]. RA patients with prior vaccination or natural immunity should be well protected from a new exposure. In cases where patients born after 1957 have not been previously vaccinated, efforts should be made to vaccinate them, keeping in mind this is a live vaccine and in general, should not be given to people actively receiving biologics or JAKi.

### Mechanisms of Vaccine Immunogenicity

Vaccines introduce an antigen to a host, which is processed by antigen-presenting cells (APCs) and presented to T cells. T cells activate B cells and provide long-term immunity by producing antibodies and generating memory T and B cells, thereby establishing both humoral and cell-mediated immunity (CMI). The novel nucleic acid vaccines developed for COVID-19 function by the same mechanism: messenger RNA (mRNA) encoding an antigen is delivered by lipid nanoparticles, and the mRNA is then translated into the protein antigen by host cells [26].

Many non-live vaccines also contain adjuvants, added substances that enhance the immune response [26]. These are often immunostimulants that mimic danger signals and activate pattern recognition receptors in APCs, releasing cytokines and co-stimulatory signals that enhance the adaptive immune response. Adjuvants can also be delivery systems, such as oil emulsions or nanoparticles, that enhance the presentation of antigens to APCs by improving their bioavailability or targeting lymphoid tissue.

Certain vaccine adjuvants are associated with local and systemic adverse events, such as injection site reactions, fever, fatigue, headache, and myalgia, which are related to reactogenicity —the manifestations of an activated immune response to the vaccine, particularly in older adults [27]. These events have been reported for AS01/AS02, AS03, and MF59 adjuvants, which are present in Shingrix (AS01) and RSV (AS01E) vaccines. However, these symptoms usually are mild to moderate and transient (e.g., 1 week or less), so they do not preclude their administration. Despite ongoing discussions about the long-term safety of vaccine adjuvants, particularly regarding aluminum-based adjuvants and neurotoxicity, evidence suggests that the adjuvants currently used in the US are safe in this respect [28].

### Challenges in Interpreting the Vaccine Literature

Creating time-tested and concise vaccination recommendations for people with RMDs, including RA, is challenging. First, new DMARDs, vaccines, and guidelines are constantly being developed. In addition to those welcomed difficulties, there is considerable heterogeneity in the vaccine study designs, which consist of (i) preclinical or lab-based immunogenicity studies that evaluate DMARD effects on vaccine immunogenicity in vitro or animal models, and (ii) human studies, which can study immunogenicity, efficacy (incidence and severity of the targeted infection), or safety outcomes.

Within immunogenicity outcomes, most studies report humoral immunity measures, such as the proportion of patients who reach a threshold antibody level (i.e. seroprotection) or a change in post-vaccination compared to pre-vaccination immunity (e.g. a four-fold increase), known as seroconversion. Cellular-mediated immunity (CMI) can also be measured and include antigen-specific T cell response. The results from humoral immunity studies do not directly translate to CMI, and vice versa. Furthermore, the outcomes can relate to the quantity (e.g., measurement of antigen-specific antibody titers or seroconversion rates) or the quality (e.g., neutralization or opsonization tests) of the immune response and the time points and thresholds defined to constitute an appropriate response can differ between studies, making comparisons between studies difficult.

Ideally, the rheumatology field would like to have recommendations based on efficacy against infection. However, most vaccine studies rarely, if ever, can evaluate clinical outcomes as primary outcomes given the logistical difficulties in achieving sufficient statistical power and the required length of follow-up. Most evidence consists of immunogenicity outcomes, which serve as a proxy for clinical outcomes. However, even for these types of studies, there is substantial heterogeneity in, for example, the length of follow-up and the definitions of what constitutes an appropriate immune response. Moreover, most infections do not have a well-established, lab-based correlate of clinical protection, meaning that the immunogenicity outcomes are an imperfect surrogate for reduction in clinical infections. The evidence is further limited if we consider studies that only evaluate patients with RA, excluding other inflammatory diseases with similar DMARD indications. In this review, we will use evidence gathered from a broader variety of RMDs plus inflammatory bowel disease (IBD) [where similar therapies to RA are often used] when there is no direct evidence from RA patients, recognizing that the underlying disease mechanisms can affect the response to vaccines, DMARDs, and their interaction.

### DMARDs Effect on Vaccine Immunogenicity

DMARDs modulate the immune response through various mechanisms, and many of them attenuate vaccine immunogenicity.

### Conventional Synthetic DMARDs (csDMARDs)

The effect of methotrexate on vaccine immunogenicity has been better elucidated than the effect of leflunomide, sulfasalazine, and hydroxychloroquine. Methotrexate inhibits multiple pathways and affects various cell types, thereby influencing both humoral and cell-mediated immune (CMI) responses [29]. Its hampering effect on vaccine immunogenicity has been demonstrated in human studies evaluating multiple vaccines in individuals with RA and other RMDs (Table 1) [30–46].

**Table 1:** Non-live vaccine immunogenicity in people with RA taking DMARDs

	Influenza	Streptococcus pneumoniae	COVID-19	Herpes zoster	HPV
csDMARDs					
Methotrexate	↓ H [36, 39] Temporarily discontinuing methotrexate (e.g., 1–2 weeks) ↑ [37, 38]	↓ H [41–45]	↓ H and CMI [33] Temporarily discontinuing methotrexate ↑ [30–32, 34]	NI	= H [35, 67, 124]
Leflunomide	= H [39]	NI, but there is a decrease in memory B cells and pneumococcal antibody levels in people with RA treated with leflunomide [49]	= H [48]	NI	NI
Sulfasalazine	↓ H in healthy volunteers [52]	NI	↑ H [50, 51]	NI	NI
HCQ	= H [53, 54]	= H [57]	= / ↑ H [48, 56, 58]	NI	NI
**bDMARDs**					
TNFi	= H [59–62, 125] ↓ H [126]	= H [60, 63, 64]	= H in people with RMD [33, 48, 65]	= H and CMI at 6 weeks, but ↓ at 1 year follow-up [66]	= H [35, 67]
Rituximab	↓ H [36, 60–62, 73–76] = CMI [73]	↓ H [60, 77–79]	↓ humoral immunity [48, 80, 81] = CMI [80, 85]	= H [84]	NI
Abatacept	↓ H [36, 87] = H [89]	↓ H [78, 79] = H [89] Decreases antibody levels, but not the opsonization index (a measure of antibody function) [127]	↓ H [48] ↓ H and CMI, but there is an improvement after the third vaccine dose [128] Temporarily discontinuing abatacept = H [91]	↓ H and CMI [88]	NI
IL inhibitors	= H and clinical [92, 93]	= H [78, 92, 94, 95]	= H [48, 96, 97]	NI	NI
**tsDMARD**					
JAKi	= / ↓ H [98] Temporarily discontinuing tofacitinib = [98]	↓ H [98] Temporarily discontinuing tofacitinib = [98]	↓ H [81, 99, 100] = H [48] Temporarily discontinuing JAKi = [91]	= H [46, 84] ↓ H [101] ↓ CMI [101]	NI

↓: decreases vaccine immunogenicity; =: does not decrease vaccine immunogenicity; ↑: improves vaccine immunogenicity; H: humoral immunity; CMI: cellular-mediated immunity; NI: no information, meaning our search did not identify any studies directly evaluating vaccine immunogenicity in users of the specific DMARD

Our search did not identify any studies evaluating RSV vaccine immunogenicity in DMARD users

RMD: rheumatic diseases; DMARDs: disease-modifying antirheumatic drugs; HPV: human papillomavirus; csDMARDs: conventional synthetic DMARDs; MCTD: mixed connective tissue disease; HCQ: hydroxychloroquine; bDMARDs: biologic DMARDs: TNFi: tumor necrosis factor inhibitors; LZV: live zoster vaccine; SpA: spondyloarthritis; RA: rheumatoid arthritis; IBD: inflammatory bowel disease; IL: interleukin; tsDMARD: targeted synthetic DMARD; JAKi: janus kinase inhibitor

**Table 2:** Immunization recommendations for immunocompromised hosts

Vaccine	Indication	Vaccine type and doses	DMARD management
**Influenza**	≥ 6 months old for all, including IC hosts	High-dose or adjuvanted vaccine	• Transiently discontinue methotrexate for 2 weeks after vaccination • Hold rituximab for at least 2 weeks after vaccination
**Pneumococcal**	> 18 years old with risk conditions*	PCV-20 or PCV-21 alone or PCV-15 followed by the 23-valent polysaccharide vaccine	• Schedule vaccination for when the next rituximab dose is due and delay rituximab for at least 2 weeks • The ACR does not recommend suspending methotrexate given the lack of direct evidence but would consider transiently discontinuing methotrexate for 1–2 weeks after vaccination based on known decreased humoral immunogenicity
**COVID-19**	≥ 6 months old for all, including IC hosts	ACIP recommendations: • unvaccinated: initial multidose series + additional dose with a 2024-25 vaccine (1 more than the general population) • previously vaccinated but not with 2024-25 vaccines: 2 doses of 2024-25 vaccines 6 months apart (1 more than the general population) • previously vaccinated with 2 doses of a 2024-25 vaccine: consider additional doses Our suggested approach, based on a high probability of natural infection with a current strain in most people: • 1 dose of a 2024-25 vaccine • additional doses in selected cases (e.g., those immunized while receiving rituximab)	• Transiently discontinue methotrexate for 1–2 weeks after vaccination • Schedule vaccination 1 week before intravenous abatacept • Transiently discontinue subcutaneous abatacept for 1–2 weeks after vaccination • Schedule vaccination approximately 4 weeks before the next planned rituximab**
**Herpes zoster**	> 18 years old IC hosts	Recombinant zoster vaccine (RZV, Shingrix)	Schedule vaccination for when the next rituximab dose is due and delay rituximab for at least 2 weeks
**HPV**	• 9–26 years old for all, including IC hosts • 27-45-year-old IC hosts and general population with shared-decision making	9-valent vaccine	Schedule vaccination for when the next rituximab dose is due and delay rituximab for at least 2 weeks
**RSV**	≥ 50 years old at increased risk of severe RSV*	• 50 to 59 years old: 1 dose of Pfizer’s Abrysvo or GSK’s Arexvy • ≥ 60 years old: 1 dose of Pfizer’s Abrysvo, GSK’s Arexvy, or Moderna’s mResvia	There are no ACR recommendations yet, but we would manage as pneumococcal or influenza immunization. The need and timing of boosters is yet to be established

ACIP: Advisory Committee for Immunization Practices; ACR: American College of Rheumatology; DMARD: Disease-modifying antirheumatic drug; IC: immunocompromised; PCV: pneumococcal conjugate vaccine; HPV: human papillomavirus; RSV: respiratory syncytial virus

* E.g., chronic lung disease and immunocompromising conditions

** From the latest ACR COVID-19 immunization guidelines, which were published before the 2024–2025 vaccines were developed

Based on high quality evidence from randomized controlled trials (RCTs), temporarily discontinuing methotrexate for one to two weeks after vaccination improves the immunogenicity of influenza and COVID-19 vaccines [30–32, 34, 37, 38]. In two RCTs [MIVAC I and II] evaluating patients with RA and psoriatic arthritis with stable disease activity and on a stable methotrexate dose, withholding methotrexate for two weeks after each of the two COVID-19 vaccine doses, and alternatively after only the second COVID-19 vaccine dose, was associated with better antibody response than continuing methotrexate [34]. In three RCTs of patients with RA receiving the seasonal influenza vaccine, withholding methotrexate resulted in an improvement in the humoral vaccine response without an increase in disease activity [37, 38, 47]. The first trial found that withholding methotrexate for four weeks before, two weeks before and two weeks after, and four weeks after was superior to continuing methotrexate [37]. Testing additional variants on how long to hold MTX, the second trial found that withholding it 2 weeks after was superior to continuing it [38]. The third study found that withholding methotrexate one week after vaccination was non-inferior to withholding it for two weeks [47].

There is less and lower-quality direct evidence as to optimal strategies to maximize vaccine response for patients receiving other csDMARDs. Leflunomide use was not associated with lower COVID-19 vaccine humoral immunogenicity compared to healthy controls in a study of patients with multiple RMD, which included RA [48]. In a study of patients with RA receiving the pandemic H1N1 influenza adjuvant-free vaccine, leflunomide use was not associated with a significantly lower humoral immune response compared to healthy controls in the adjusted analysis [39]. However, in people with RA taking leflunomide, there is a decrease in memory B cells and pneumococcal antibody levels [48, 49]. In the case of sulfasalazine and hydroxychloroquine, a more nuanced relationship appears to exist, where the specific vaccine and the underlying RMD were related to vaccine immunogenicity. Overall, except for methotrexate, and perhaps leflunomide, there is limited evidence that hydroxychloroquine and sulfasalazine impair vaccine immunogenicity, and no recommendation exists to hold leflunomide, hydroxychloroquine, or sulfasalazine at the time of vaccine administration [50–58].

### Biologic DMARDs (bDMARDs)

#### TNF Inhibitors

TNF-alpha inhibition has broad anti-inflammatory effects, including the prevention of matrix degradation which produces proinflammatory molecules and the inhibition of leukocyte activation [29]. Despite TNF inhibitors’ wide-ranging mechanisms of action, they do not appear to meaningfully decrease vaccine immunogenicity in people with RMD. Multiple studies evaluating influenza, pneumococcal, COVID-19, VZV, and HPV vaccines have found that people with RMD who use TNF inhibitors (TNFi) do not have significantly lower vaccine humoral immune responses compared to controls [33, 35, 48, 59–67]. However, there is less evidence evaluating CMI. In the case of VZV, a study evaluating the live attenuated VZV vaccine (ZVL, Zostavax) found that the humoral immune response in TNFi users was robust. However, the CMI response was not significantly different between individuals who received Zostavax and controls who received placebo at six weeks, and it had returned to baseline levels by one-year follow-up [66]. In contrast to RMD patients, it is also noteworthy that studies evaluating patients with IBD show that TNFi users have a significant decrease in humoral vaccine immunogenicity compared to non-TNFi users [68–72], although whether that observation is due to the TNFi use, the disease itself, or other co-therapies (e.g., higher dose glucorticoid use) is unclear.

#### Rituximab

Among all the DMARDs approved for RA treatment, rituximab appears to have the largest immune-hampering effect to vaccine response. Rituximab depletes B cells by binding CD20, inhibiting antigen presentation and antibody production [29]. Multiple studies evaluating influenza, pneumococcal, and COVID-19 vaccines have shown that the humoral vaccine immune response in rituximab users is significantly decreased [36, 48, 60–62, 73–81]. In four prospective studies, immunogenicity was greater after influenza vaccination when the vaccine was administered approximately six months (range: five to 10 months) after rituximab [73, 76, 82, 83]. An exception is a study assessing the safety and immunogenicity of the recombinant, adjuvanted VZV vaccine (RZV, Shingrix), which found that rituximab users had preserved humoral immunogenicity [84].

Fewer studies have evaluated CMI markers in rituximab users, but it appears like it might be less impaired than the humoral response. In two COVID-19 and one influenza study, unlike the humoral response, rituximab users did not have a significantly lower CMI response compared to healthy controls or individuals treated with other DMARDs [73, 80, 85]. In another study evaluating the COVID-19 vaccines in rituximab users, the CMI response was found to be independent of the humoral response [86].

#### Abatacept

Abatacept binds CD80 and CD86, blocking T-cell co-stimulation and activation [29]. Vaccine immunogenicity has been evaluated in abatacept users for influenza, pneumococcal, COVID-19, and VZV vaccines but not for HPV or RSV. In most of these studies, abatacept users exhibited a decreased humoral and CMI response [36, 48, 78, 79, 87, 88]. However, abatacept users have also been shown to be able to mount an adequate (even if reduced) humoral immune response following vaccination [89, 90]. In a randomized controlled platform trial that studied multiple therapies under a master protocol (the COvid VaccinE Response [COVER] trial) evaluating the humoral immune response to a COVID-19 vaccine booster in patients with RA and psoriatic arthritis, abatacept use was associated with a lower response to a COVID-19 booster compared to use of TNFi or IL-17 therapies, but holding abatacept for 2 weeks after vaccination was not associated with a better immune response compared to continuing treatment [91].

#### Interleukin (IL) Inhibitors (IL-6Ri and IL-1Ri)

IL-6 receptor blockers inhibit B cell differentiation and the activation of leukocytes. Consistently, their use has not been associated with a decrease in humoral vaccine immunogenicity in studies evaluating patients’ response to influenza, pneumococcal, and COVID-19 vaccines [48, 78, 92–95].

IL-1 receptor inhibitors prevent the activation of leukocytes by blocking the binding of IL-1 to its receptors. Studies evaluating the response to the COVID-19 vaccine among people with autoinflammatory diseases found no evidence of a decrease in humoral vaccine immunogenicity [96, 97]. Currently, we lack studies evaluating the response to other vaccines.

### Targeted Synthetic DMARDs

#### JAK Inhibitors

Baricitinib, tofacitinib, and upadacitinib block leukocyte maturation by inhibiting the JAK-STAT pathway [29]. In studies of JAKi users, the vaccine immunogenicity results are heterogeneous and appear to depend on the specific vaccine.

In the case of influenza, vaccine immunogenicity in JAKi users appears to be sufficient but attenuated. In a study evaluating the influenza vaccine in RA patients, there was no difference in the number of tofacitinib and placebo-using patients who achieved a satisfactory humoral response. However, fewer tofacitinib users achieved protective antibody titers [98]. For pneumococcus and COVID-19, multiple studies have shown that JAKi users have decreased humoral immune responses [81, 91, 98–100]. However, based on the COVER trial, COVID-19 vaccine humoral immunity was not superior when JAKi was temporarily discontinued for 2 weeks after vaccination compared to continuing it, and was associated with a significantly increased risk of disease flare [91].

The immunogenicity of the VZV vaccine in JAKi users is particularly important, as these patients are more vulnerable to viral reactivation than users of other DMARDs [16, 17]. JAKi decrease type I and II interferon activity, and interferons are critical in suppressing latent VZV and preventing viral reactivation. The CMI response to Shingrix appears to be decreased in JAKi users, consistent with their mechanism of action [101, 102]. It is less clear if the humoral response to VZV vaccination is diminished [84, 101].

### Disease-Specific Vaccine Recommendations

The American College of Rheumatology (ACR) published its latest guideline for vaccinations in 2022 [103]. In it, the ACR task force recommended vaccine use and suggested timing considerations with respect to vaccination and DMARD use for children and adults with RMD. They mainly were intended for use by rheumatology providers in the US, given the global variability in vaccine availability and the epidemiology of infectious diseases. However, they are recognized as useful for providers in other countries. Given that the new pneumococcal conjugate vaccines (PCV15, PCV20, and PCV21) and the RSV vaccine were not yet approved at the time the guideline was developed, they were not included in the guideline’s literature search. Similarly, COVID-19 vaccination recommendations were not included in this main guideline, as the pandemic was ongoing at the time and evidence was still emerging. The ACR developed separate recommendations for COVID-19, with the latest (5th ) vaccination guideline published in 2022 [104], which now aligns well with current CDC ACIP recommendations for COVID-19 vaccination in immunocompromised individuals [105]. 

The adult and pediatric immunization schedules established by the CDC are based on recommendations from the Advisory Committee on Immunization Practices (ACIP), a federal advisory committee constituted by medical and public health experts. The ACIP regularly reviews vaccine literature and provides guidance based on the latest evidence and developments [106]. Before recommendations are published, any updates to their recommendations are listed by the CDC in their Morbidity and Mortality Weekly Report.

#### Influenza Virus

Influenza vaccines are of three main types: live attenuated influenza vaccines, recombinant influenza vaccines, and inactivated influenza vaccines. Inactivated influenza vaccines can be further categorized as standard-dose or high-dose depending on the amount of influenza hemagglutinin antigen present per vaccine dose and as adjuvanted when the vaccine contains an additional immunogenic ingredient. The vaccines are also classified based on their valency, which refers to the number of hemagglutinin antigens present, typically three (trivalent vaccines) or four (quadrivalent vaccines) [107].

The CDC’s ACIP recommends yearly vaccination for everyone six months and older with any vaccine for which they have no contraindications (e.g., avoiding live attenuated vaccines in immunocompromised individuals) but emphasizes the importance of vaccination in people at increased risk for severe illness and complications, such as immunocompromised individuals [107]. They also recommend using a high-dose or adjuvanted vaccine for people 65 years and older and for immunocompromised individuals of any age, which aligns with the ACR vaccination guideline recommendations [103].

The ACR guideline also addresses the management of DMARDs around the time of influenza vaccination. Based on the RCT evidence described above, and if disease activity allows because the patient is doing well clinically, they recommend temporarily discontinuing methotrexate for two weeks after immunization [103]. All other immunosuppressive medications besides rituximab can be continued around the time of vaccination. Rituximab presents a unique scenario given its 6-month dosing interval. While in the ACR guidelines, many vaccines are recommended to be timed carefully in relation to rituximab dosing, influenza is an exception given that an annual, seasonal vaccine was deemed impractical to time in relation to rituximab dosing, and there was no recommendation to hold or otherwise time influenza vaccination with consideration of the rituximab dosing schedule.

#### Streptococcus Pneumoniae

Five pneumococcal vaccines are available in the US: four pneumococcal conjugate vaccines (PCV) and one pneumococcal polysaccharide vaccine [108]. PCVs induce a more robust and durable response than the polysaccharide vaccine and are, therefore, generally preferred. The four PCVs available are the 13-valent pneumococcal conjugate vaccine (PCV13), the 15-valent pneumococcal conjugate vaccine (PCV15), the 20-valent pneumococcal conjugate vaccine (PCV20), and the newest, the 21-valent pneumococcal conjugate vaccine (PCV21). The polysaccharide vaccine is a 23-valent polysaccharide vaccine (PPSV23). For all of them, the valency denotes the number of bacterial serotypes it protects against.

The ACIP recommends vaccination with a 20- or 21-valent PCV alone or a 15-valent PCV combined with the PPSV23 for all PCV naïve people who are 50 years of age or older or are between the ages of 19 and 49 but have a high-risk condition, which includes chronic lung disease or an immunocompromising condition including RA (Table 2) [108]. The 50-year-old cutoff for vaccination in people without high-risk conditions is a recent modification to the previous 65-year-old cutoff, stemming from a desire to improve vaccination coverage and reduce disease incidence and mortality in adults aged 50 to 64. The ACR guidelines recommend vaccination of all adults age 18 years and older with RMD who are taking immunosuppressive medications [103].

Unlike with influenza vaccines, the ACR guidelines do not recommend suspending methotrexate around the time of pneumococcal vaccination as there were no trial data published addressing the question as to whether temporarily holding methotrexate would assist immune response to pneumococcal vaccines. However, given the number of studies showing reduced pneumococcal responses in those taking methotrexate and given the trial data with influenza and COVID-19 vaccines showing a clear improvement in humoral response if methotrexate is held for one to two weeks, we believe it is reasonable to also hold methotrexate dosing for one to two weeks at the time of pneumococcal vaccination. People receiving rituximab are advised to time the vaccination for when the next dose is due (i.e., > 6 months since the most recent dose) and not administer rituximab for at least two weeks after vaccination. Other DMARDs can be continued without interruption.

#### SARS-CoV-2

Two types of COVID-19 vaccines are recommended in the US: mRNA vaccines and a protein subunit vaccine. The currently authorized mRNA vaccines are the 2024–2025 Moderna COVID-19 vaccine (Spikevax) and the 2024–2025 Pfizer-BioNTech COVID-19 vaccine (Comirnaty), both of which are approved for use starting at six months of age. The protein subunit vaccine is the 2024–2025 Novavax COVID-19 vaccine, approved beginning at 12 years of age [105].

In 2024, the ACIP released two reports with recommendations for COVID-19 vaccination in individuals aged six months and older, as well as in older adults and those with moderate or severe immunocompromising conditions [109, 110]. These recommendations addressed new strains and the known waning effectiveness of the COVID-19 vaccine over time. Vaccination with any current (e.g., 2024–2025) COVID vaccine is currently recommended for all persons aged six months and older, but the number of doses depends on the immunosuppression status of the receiver, their age, the number of doses they have previously received, whether they received a multidose initial series, and if any of these doses were a 2024-25 vaccine.

The ACIP’s recommendations for moderately or severely immunosuppressed people are as follows: (i) for those who are unvaccinated or have not completed an initial series, they should receive a multidose initial series and an additional dose six months after the initial series is completed (minimum interval of two months) with any of the approved 2024-25 vaccines; (ii) for those who have previously completed an initial series, they should receive two vaccine doses at least eight weeks after the initial series is completed and at least six months apart with any of the three approved 2024-25 vaccines; for those who have received two or more doses of a 2024-25 vaccine, additional doses should be discussed and administered at least eight weeks after the last dose, after shared decision making. However, it is unclear how many additional doses are required or whether individuals who have received multiple vaccine doses or have had natural infection(s) with a current strain would significantly benefit from additional doses of the current vaccines. Our suggested approach is that everyone receives one dose of a 2024-25 vaccine and additional doses be considered only in selected cases, e.g., in individuals who were vaccinated while receiving rituximab or other therapies known to impair vaccine response (e.g. mycophenolate mofetil, which might be used in RA patients if they have concomitant ILD).

In May 2025, the US Food and Drug Administration’s (FDA) Vaccines and Related Biological Products Advisory Committee met to discuss the 2025–2026 COVID-19 vaccines. Following this meeting, they have advised the approved vaccine manufacturers that the 2025–2026 vaccines to be used in the US, starting in the fall of 2025, should be monovalent JN.1-lineage-based vaccines. The ACIP is expected to meet in June 2025 to discuss updated immunization recommendations. It is anticipated that they will discuss whether COVID-19 vaccination recommendations should continue to be universal or if they should be only risk-based.

ACR released its latest guidelines for COVID-19 vaccination in 2022, so they are based on the initial vaccines and not the 2024-25 vaccines referred to by the ACIP guidelines [104]. While separate COVID-19 guidance recommendations are no longer being updated, given the increasing convergence over time with the CDC’s ACIP recommendations for immunocompromised patients, the most recent ACR guidance does include recommendations regarding the management of DMARDs around the time of COVID-19 vaccination and boosting. For rituximab users, vaccination is recommended to be scheduled approximately four weeks before the next planned rituximab cycle or based on the measurement of CD19 B cells. Methotrexate should be discontinued for one to two weeks after vaccination. For abatacept users, the recommendations are to schedule vaccinations one week before intravenous abatacept or to hold subcutaneous abatacept for one to two weeks after a vaccine dose. However, the COVER trial, which succeeded the ACR guidelines, specifically evaluated the immunogenicity of COVID-19 vaccine boosters and found that suspending abatacept (and JAKi, TNFi, and IL-17 therapies) did not significantly improve immunogenicity and was associated with an increased risk for disease flares in patients with RA and psoriatic arthritis [91].

#### Varicella Zoster Virus

Two types of VZV vaccines are available in the world: the live attenuated vaccine (ZVL, Zostavax) and the recombinant vaccine (RZV, Shingrix). Since late 2020, Zostavax is no longer marketed in the US, as Shingrix appears to be more effective and longer-lasting. In the general population, the effectiveness of Shingrix is high, with an approximate 80–90% clinical effectiveness. However, it is likely lower and of somewhat shorter duration (yet still conferring long-term protection) in patients with RA [111]. An important practical issue is the risk for reactogenicity with Shingrix. Due to its potent AS01B adjuvant, the risk of severe (grade 3) reactogenicity is reasonably high (approximately 15%), primarily manifesting as low-grade fever, myalgias, and arthralgias, and may be mistaken for a disease flare. However, reactogenicity symptoms resolve within several days or one week, unlike a disease flare that may persist for longer. Thus, patients should be counseled about this risk and told that they can take antipyretics and analgesics after their vaccination. If symptoms persist beyond one week, the patient should be assessed for potential RA disease flare that may require a change in RA medications.

The recommendations from the ACIP and ACR for people with RMD taking immunosuppressive medications and vaccination are comparable, stating that patients should receive Shingrix starting at age 18 [103, 112]. One yet-unknown consideration is when or if Shingrix should be re-administered based on waning immunity, particularly CMI. For non-immunocompromised individuals, the ACIP recommends vaccination at 50 and older. Similar to the pneumococcal vaccines, the ACR recommendation for rituximab users is to administer vaccination at the time rituximab is due and to delay rituximab for at least two weeks after vaccination [103].

Despite its lower immunogenicity and safety concerns for live vaccines in immunosuppressed patients, Zostavax could be used in countries where the recombinant vaccine is unavailable. The ACR guideline recommends deferring live vaccines in patients with RMD taking immunosuppressive medications, given the theoretical concern for disease and complications related to the vaccine strain. However, Zostavax appears safe in selected patients. In an RCT evaluating the safety and immunogenicity of TNFi in patients using them with or without methotrexate and prednisone, there were no cases of VZV infection or reactivation [66]. In a second trial studying 55 RA patients starting tofacitinib and methotrexate, there was one case of cutaneous vaccine dissemination in a methotrexate user who lacked primary immunity to varicella, a contraindication for Zostavax [46]. Lastly, there were no cases of shingles after vaccination in a large observational study of patients who were inadvertently vaccinated while on bDMARDs [113]. Taken together, these data suggest that if necessary and despite the safety concern of using live virus vaccines in immunocompromised individuals, the live zoster vaccine (and perhaps other live virus vaccines) might be given safely to TNFi and other cytokine inhibitor users (but perhaps not JAKi users) in circumstances where vaccination is a high priority, particularly if patients are unable or unwilling to stop their biologic for several weeks, receive vaccination, wait approximately four additional weeks, and then resume biologic therapy.

#### Human Papillomavirus

There are three types of HPV vaccines licensed for use in the US: the bivalent (Cervarix), quadrivalent (Gardasil), and 9-valent vaccines (Gardasil 9). However, since 2016, only the 9-valent vaccine has been distributed [114]. The bivalent vaccine targets HPV types 16 and 18, the two types responsible for most cervical cancers. The quadrivalent vaccine targets types 6 and 11, which cause the majority of genital warts, in addition to types 16 and 18. The 9-valent vaccine includes types 31, 33, 45, 52, and 58, which are also oncogenic, as well as all the previous types.

The ACIP and ACR recommendations regarding HPV vaccination coincide. ACIP recommends routine vaccination at age 11 or 12, noting that the vaccine can be given as early as age nine [114]. The vaccine is meant to be used prior to sexual maturity, as HPV infection is common with sexual intercourse. For unvaccinated people, catch-up vaccination can be offered until age 26, particularly for those who have had few or no sexual partners by that age. Between the ages of 27 and 45, they recommend catch-up vaccination after shared decision-making. The ACR guideline recommends considering catch-up vaccination on a case-by-case basis for people with RMD who are in this age group and take immunosuppressive medications [103]. Like with the pneumococcal and VZV vaccine, for rituximab users, vaccination should be timed around when the next dose is due, and rituximab should be delayed for at least two weeks. All other immunosuppressive drugs, including methotrexate, should be continued without changes.

#### Respiratory Syncytial Virus

There are three RSV vaccines available in the US: one mRNA vaccine (Moderna’s mResvia) and two protein subunit vaccines: GSK’s adjuvanted vaccine, Arexvy, and Pfizer’s bivalent recombinant vaccine, Abrysvo [115]. Abrysvo was initially approved for use in pregnant persons to prevent RSV in infants and is still the only RSV vaccine approved for use during pregnancy. In 2023, Abrysvo and Arexvy were first approved for use in adults 60 years and older to prevent RSV lower respiratory tract disease, followed by the approval of mResvia for the same population in December 2024.

Following the initial approval of adult RSV vaccines, the ACIP developed the 2024 recommendations to vaccinate the general population at 75 years and older and individuals at high risk of severe RSV disease at 60 to 74 years old with a single vaccine dose [115]. The definition of people at increased risk of RSV includes people with congestive heart disease, chronic obstructive pulmonary disease, or other chronic lung disease, like ILD, and individuals with immunocompromising conditions (e.g., RA).

Soon after the initial ACIP guidelines, the US FDA expanded the licenses of Abrysvo and Arexvy to include individuals at increased risk between the ages of 18 and 59 for Abrysvo and between ages 50 and 59 for Arexvy. The ACIP has now expanded its recommendation to include the vaccination of adults between 50 and 59 years who are at increased risk of severe RSV disease.

The current ACIP recommendation is that individuals who have already received a dose of an RSV vaccine do not require additional doses. However, it is anticipated that additional doses will be needed as the vaccine efficacy wanes over time. In the general population, the vaccine efficacy against lower respiratory tract disease is approximately 81% in the first RSV season after vaccination but decreases to approximately 61% in the second season [116]. Although there is no direct evidence evaluating RSV vaccine immunogenicity longitudinally in RA patients using DMARDs, it is logical to assume that the same pattern will be observed and that vaccination every approximately two years may be warranted.

Since immunization for RSV in adults is a new recommendation, there are no studies evaluating the vaccines’ immunogenicity in DMARD users. Given its recent availability, the ACR guideline does not include recommendations for the use of RSV nor management of DMARDs around the time of vaccination, but it is likely that recommendations would mirror the vaccine guidance for pneumococcal or influenza vaccination.

#### Measles, Mumps, and Rubella

Two measles, mumps, and rubella (MMR) vaccines are available in the US: the M-M-R-II vaccine and the PRIORIX vaccine, both live attenuated vaccines [117]. The ACR vaccination guideline has two recommendations regarding live vaccines [103]. First, they conditionally recommend deferring them for patients with RMD taking immunosuppressive medications, and second, if a live virus vaccine must be given, they conditionally recommend holding immunosuppressive medications before and four weeks after live attenuated vaccination for patients with RMD. These recommendations stem from safety concerns regarding local or disseminated disease related to the vaccine strain in immunocompromised hosts.

There are three observational studies evaluating MMR safety among patients with RMD. In two large cohort studies, one involving juvenile idiopathic arthritis (JIA) patients and the other pediatric patients with RMD, no cases of measles, mumps, or rubella were reported after MMR vaccination [118, 119]. However, in a third observational study with only seven patients who received MMR vaccination, a patient with systemic JIA receiving canakinumab developed pneumonia a week after immunization [120]. The investigators could not completely rule out that the infection was related to the measles strain introduced by the vaccine, but they considered it unlikely.

For the general population, the ACIP guidelines recommend routine MMR vaccination with two doses of any of the two vaccines, the first at 12 to 15 months of age and the second at four to six years of age [121]. For adults and teenagers, vaccination is recommended for those without known or presumptive evidence of immunity to measles, mumps, or rubella. Presumptive evidence of immunity can be established by a prior history of vaccination, laboratory evidence of immunity (IgG in serum), laboratory confirmation of disease, or it is assumed in those born before 1957. For those lacking prior vaccination or presumptive immunity, vaccination should be given with two doses separated by at least 28 days, as two vaccine doses are 97% (range 67–100%) effective at preventing measles. However, one dose has 93% (range 39–100%) effectiveness [122]. There is currently no recommendation to administer a booster dose to prevent measles in those with prior vaccination or presumptive immunity. One exception would be health care or other workers with potential measles exposure in the midst of an outbreak. In such cases, a titer could be checked and a booster given as needed.

If an exposure occurs, post-exposure prophylaxis (PEP) for measles should be offered to people who do not have presumptive evidence of immunity. PEP can be administered as an MMR vaccine if it is given within 72 h of exposure or as an immunoglobulin if it is administered within six days of exposure. They should not be administered together, as the immunoglobulin renders the vaccine ineffective. Immunoglobulins are considered safe for use in individuals who are immunocompromised. However, they do not provide long-term protection [123].

## Conclusions

Many of the infections causing significant morbidity and mortality in people with RA are potentially preventable with safe and effective vaccines. The current CDC and ACR guidelines are good resources for staying up to date with the latest immunization recommendations, which constantly evolve as new DMARDs and vaccines are developed and with the commitment of the ACR to maintain ‘living guidelines’ and routinely undergo periodic updates as needed. The vaccine field would benefit from more homogeneous immunogenicity outcomes and, whenever possible, to collect data on clinical outcomes. We can achieve the latter by using increasingly accessible real-world data to overcome the logistical limitations of large and lengthy clinical studies. We need studies evaluating the short- and long-term effectiveness of the new RSV and COVID-19 vaccines in DMARD users, as well as more studies evaluating established vaccines where there are still knowledge gaps. Understanding the optimal way of vaccinating individuals using various types of DMARDs remains a highly unmet need.

## Data Availability

No datasets were generated or analysed during the current study.

## Key References

The ACR vaccination guidelines for patients with RMD are a valuable resource for immunization indications and guidance on DMARDs management around the time of vaccination.

1. Bass AR, Chakravarty E, Akl EA, Bingham CO, Calabrese L, Cappelli LC, et al. 2022 American College of Rheumatology Guideline for Vaccinations in Patients With Rheumatic and Musculoskeletal Diseases. Arthritis Rheumatol. 2023;75(3):333-48.

The ACIP vaccination guidelines are frequently updated and contain the latest immunization evidence. These are the most recent reports for HPV, herpes zoster, influenza, RSV, COVID-19, and pneumococcus.

2. Meites E, Szilagyi PG, Chesson HW, Unger ER, Romero JR, Markowitz LE. Human Papillomavirus Vaccination for Adults: Updated Recommendations of the Advisory Committee on Immunization Practices. MMWR Morb Mortal Wkly Rep. 2019;68(32):698-702.
3. Anderson TC, Masters NB, Guo A, Shepersky L, Leidner AJ, Lee GM, et al. Use of Recombinant Zoster Vaccine in Immunocompromised Adults Aged ≥19 Years: Recommendations of the Advisory Committee on Immunization Practices - United States, 2022. MMWR Morb Mortal Wkly Rep. 2022;71(3):80-4.
4. Grohskopf LA, Ferdinands JM, Blanton LH, Broder KR, Loehr J. Prevention and Control of Seasonal Influenza with Vaccines: Recommendations of the Advisory Committee on Immunization Practices - United States, 2024-25 Influenza Season. MMWR Recomm Rep. 2024;73(5):1-25.
5. Britton A, Roper LE, Kotton CN, Hutton DW, Fleming-Dutra KE, Godfrey M, et al. Use of Respiratory Syncytial Virus Vaccines in Adults Aged ≥60 Years: Updated Recommendations of the Advisory Committee on Immunization Practices - United States, 2024. MMWR Morb Mortal Wkly Rep. 2024;73(32):696-702.
6. Panagiotakopoulos L, Moulia DL, Godfrey M, Link-Gelles R, Roper L, Havers FP, et al. Use of COVID-19 Vaccines for Persons Aged ≥6 Months: Recommendations of the Advisory Committee on Immunization Practices - United States, 2024-2025. MMWR Morb Mortal Wkly Rep. 2024;73(37):819-24.
7. Lauren E. Roper MG, Ruth Link-Gelles, Danielle L. Moulia, Christopher A. Taylor, Georgina Peacock, Noel Brewer, Oliver Brooks, George Kuchel, H. Keipp Talbot, Robert Schechter, Katherine E. Fleming-Dutra, Lakshmi Panagiotakopoulos. Use of Additional Doses of 2024–2025 COVID-19 Vaccine for Adults Aged ≥65 Years and Persons Aged ≥6 Months with Moderate or Severe Immunocompromise: Recommendations of the Advisory Committee on Immunization Practices — United States, 2024. MMWR Morb Mortal Wkly Rep. 2024(73):1118–23.
8. Kobayashi M, Leidner AJ, Gierke R, Xing W, Accorsi E, Moro P, et al. Expanded Recommendations for Use of Pneumococcal Conjugate Vaccines Among Adults Aged ≥50 Years: Recommendations of the Advisory Committee on Immunization Practices - United States, 2024. MMWR Morb Mortal Wkly Rep. 2025;74(1):1-8.
